# Tetra-μ-2-methyl­benzoato-κ^8^ *O*:*O*′-bis­[(ethanol-κ*O*)copper(II)]

**DOI:** 10.1107/S1600536811042322

**Published:** 2011-10-22

**Authors:** Sheng-Liang Ni, Jie-Lian Yu, Yuan-Ling Wang, Jian-Li Lin

**Affiliations:** a Department of Chemistry, Huzhou Teachers College, Huzhou, Zhejiang 313000, People’s Republic of China; b Center of Applied Solid State Chemistry Research, Ningbo University, Ningbo, 315211 People’s Republic of China

## Abstract

In the title dinuclear complex, [Cu_2_(C_8_H_7_O_2_)_4_(C_2_H_5_OH)_2_], four 2-methyl­benzoato anions form a cage around two Cu^II^ ions in a *syn–anti* configuration. Two ethanol mol­ecules coordinate the Cu atoms in apical positions, giving an overall square-pyramidal coordination geometry. The Cu⋯Cu separation is 2.600 (1) Å. In the crystal, mol­ecules are assembled into chains extending in [001] through O—H⋯O hydrogen bonds.

## Related literature

For the crystal stuctures of related complexes, see: Melnik *et al.* (1984 ▶); Sunil *et al.* (2008 ▶); Danish *et al.* (2010 ▶).
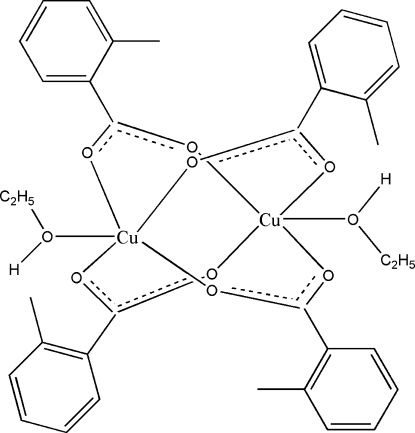


## Experimental

### 

#### Crystal data

[Cu_2_(C_8_H_7_O_2_)_4_(C_2_H_6_O)_2_]
*M* *_r_* = 759.76
Triclinic, 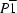

*a* = 10.989 (2) Å
*b* = 12.369 (3) Å
*c* = 14.143 (3) Å
α = 66.58 (3)°
β = 87.79 (3)°
γ = 85.46 (3)°
*V* = 1758.4 (7) Å^3^
*Z* = 2
Mo *K*α radiation
μ = 1.27 mm^−1^
*T* = 298 K
0.28 × 0.12 × 0.10 mm

#### Data collection

Rigaku R-AXIS RAPID CCD diffractometer
Absorption correction: multi-scan (*ABSCOR*; Higashi, 1995 ▶) *T*_min_ = 0.831, *T*_max_ = 0.886
17320 measured reflections
7931 independent reflections
4219 reflections with *I* > 2σ(*I*)
*R*_int_ = 0.044

#### Refinement

*R*[*F* ^2^ > 2σ(*F* ^2^)] = 0.050
*wR*(*F* ^2^) = 0.166
*S* = 1.15
7931 reflections
433 parameters
H-atom parameters constrained
Δρ_max_ = 1.54 e Å^−3^
Δρ_min_ = −1.64 e Å^−3^

### 

Data collection: *RAPID-AUTO* (Rigaku, 1998 ▶); cell refinement: *RAPID-AUTO*; data reduction: *CrystalStructure* (Rigaku/MSC, 2004 ▶); program(s) used to solve structure: *SHELXS97* (Sheldrick, 2008 ▶); program(s) used to refine structure: *SHELXL97* (Sheldrick, 2008 ▶); molecular graphics: *SHELXTL* (Sheldrick, 2008 ▶); software used to prepare material for publication: *SHELXTL*.

## Supplementary Material

Crystal structure: contains datablock(s) global, I. DOI: 10.1107/S1600536811042322/cv5148sup1.cif

Structure factors: contains datablock(s) I. DOI: 10.1107/S1600536811042322/cv5148Isup2.hkl

Additional supplementary materials:  crystallographic information; 3D view; checkCIF report

## Figures and Tables

**Table 1:** Hydrogen-bond geometry (Å, °)

*D*—H⋯*A*	*D*—H	H⋯*A*	*D*⋯*A*	*D*—H⋯*A*
O9—H91⋯O5^i^	0.85	2.04	2.841 (6)	156
O10—H101⋯O4^ii^	0.86	2.00	2.831 (6)	162

Symmetry codes: (i) 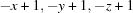; (ii) 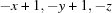.

**Fig. 1.**
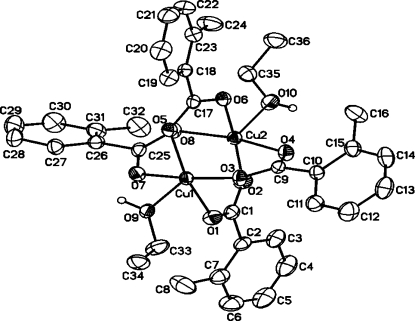
ORTEP view of the title compound with atomic numbering. The dispalcement ellipsoids are drawn at 45% probability level. C-bound H atoms omitted for clarity.

**Fig. 2.**
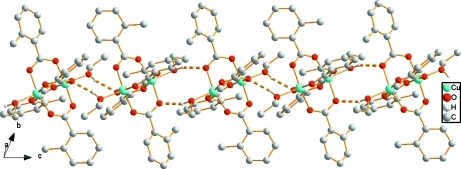
A portion of the crystal packing showing hydrogen–bonded (dashed lines) chain. C-bound H atoms omitted for clarity.

## supplementary crystallographic information

### Comment

Recently, a number of crystal structures of dinuclear Cu complexes with 2–methylbenzoic acid were published, for example, tetrakis(*µ*–2–methylbenzoato–κ^2^*O:O'*)–bis [(methanol–κ*O*)copper(II)] (II)(Danish *et al.*,2010) and tetrakis(*µ*–2–methylbenzoato–κ^2^*O:O'*)–bis [(2–methylbenzoic acid–κ*O*)copper(II)] (III) (Sunil *et al.*, 2008) are among others [see (Melnik *et al.*, 1984)]. Herewith we present the crystal of the title compound (I), a new dinuclear Cu complex with 2–methylbenzoic acid.

In (I) (Fig. 1), four 2–methylbenzoate ligands form a cage around two Cu atoms in a *syn-anti* configuration. The Cu1···Cu2 separation is 2.600 (1) Å, the Cu—O bond lengths of the cage carboxylates vary in 1.928 (4) – 2.017 (4) Å. The ethanol coordinating bond lengths [Cu1—O9 of 2.149 (4) Å and Cu2—O10 of 2.161 (4) Å)] are normal, though slightly different from those observed in (II), where Cu centers are coordinated by methanol.

In the crystal structure, the molecules are assembled into one-dimensional chains extending in [001] through O—H···O hydrogen bonds.

### Experimental

Freshly prepared CuCO_3_ was essential for an optimal synthysis. At first, 1.0 cm^3^ (1 *M*) aqueous Na_2_CO_3_ was dropwise added to a stirred aqueous solution of (0.2490 g, 1.0 mmol) CuSO_4_.5H_2_O in 4 cm^3^ of H_2_O, the produced a blue precipitate, Cu(OH) _2–2x_(CO_3_)*_x_*. *y*H_2_O, which was centrifuged and washed with doubly distilled water until no SO_4_^-2^ anions were detected in the supernatant. The freshly blue precipitate was subsequently added to a stirred solution of 2–methyl benzoic acid (0.5450 g, 4.0 mmol) in 20 cm^3^ C_2_H_5_OH–H_2_O (1:1,*v*/*v*). The mixture was stirred for 1 h and filtered. Insoluble solid was then filtered out, the resulting blue filtrate (pH = 4.80) was allowed to stand at room temperature, and green block crystals were obtained by slow evaporation for some days (yield:42%).

### Refinement

All H–atoms bonded to C were positioned geometrically and refined using a riding model, with d(C–H) = 0.93-0.96 Å and *U*_iso_(H) = 1.2-1.5 *U*_eq_(C). H atoms attached to O atoms were found in a difference Fourier map and were refined using a riding model, with the O–H distances fixed as initially found and with *U*_iso_(H) values set at 1.2 *U*eq(O).

### Crystal data

**Table:**

[Cu_2_(C_8_H_7_O_2_)_4_(C_2_H_6_O)_2_]	*Z* = 2
*M**_r_* = 759.76	*F*(000) = 788
Triclinic, *P*1	*D*_x_ = 1.435 Mg m^−^^3^
Hall symbol: -P 1	Mo *K*α radiation, λ = 0.71073 Å
*a* = 10.989 (2) Å	Cell parameters from 25 reflections
*b* = 12.369 (3) Å	θ = 3.1–27.5°
*c* = 14.143 (3) Å	µ = 1.27 mm^−^^1^
α = 66.58 (3)°	*T* = 298 K
β = 87.79 (3)°	Block, green
γ = 85.46 (3)°	0.28 × 0.12 × 0.10 mm
*V* = 1758.4 (7) Å^3^	

### Data collection

**Table:**

Rigaku R-AXIS RAPID CCD diffractometer	7931 independent reflections
Radiation source: fine-focus sealed tube	4219 reflections with *I* > 2σ(*I*)
graphite	*R*_int_ = 0.044
ω scans	θ_max_ = 27.5°, θ_min_ = 3.1°
Absorption correction: multi-scan (*ABSCOR*; Higashi, 1995)	*h* = −14→14
*T*_min_ = 0.831, *T*_max_ = 0.886	*k* = −14→16
17320 measured reflections	*l* = −18→18

### Refinement

**Table:**

Refinement on *F*^2^	Primary atom site location: structure-invariant direct methods
Least-squares matrix: full	Secondary atom site location: difference Fourier map
*R*[*F*^2^ > 2σ(*F*^2^)] = 0.050	Hydrogen site location: inferred from neighbouring sites
*wR*(*F*^2^) = 0.166	H-atom parameters constrained
*S* = 1.15	*w* = 1/[σ^2^(*F*_o_^2^) + (0.0292*P*)^2^ + 5.9014*P*] where *P* = (*F*_o_^2^ + 2*F*_c_^2^)/3
7931 reflections	(Δ/σ)_max_ < 0.001
433 parameters	Δρ_max_ = 1.54 e Å^−^^3^
0 restraints	Δρ_min_ = −1.64 e Å^−^^3^

### Special details

**Table:**

Geometry. All e.s.d.'s (except the e.s.d. in the dihedral angle between two l.s. planes) are estimated using the full covariance matrix. The cell e.s.d.'s are taken into account individually in the estimation of e.s.d.'s in distances, angles and torsion angles; correlations between e.s.d.'s in cell parameters are only used when they are defined by crystal symmetry. An approximate (isotropic) treatment of cell e.s.d.'s is used for estimating e.s.d.'s involving l.s. planes.
Refinement. Refinement of *F*^2^ against ALL reflections. The weighted *R*-factor *wR* and goodness of fit *S* are based on *F*^2^, conventional *R*-factors *R* are based on *F*, with *F* set to zero for negative *F*^2^. The threshold expression of *F*^2^ > σ(*F*^2^) is used only for calculating *R*-factors(gt) *etc*. and is not relevant to the choice of reflections for refinement. *R*-factors based on *F*^2^ are statistically about twice as large as those based on *F*, and *R*- factors based on ALL data will be even larger.

### Fractional atomic coordinates and isotropic or equivalent isotropic displacement parameters (Å^2^)

**Table:**

	*x*	*y*	*z*	*U*_iso_*/*U*_eq_	
Cu1	0.58348 (6)	0.50009 (6)	0.33035 (5)	0.03815 (19)	
Cu2	0.46734 (6)	0.46678 (6)	0.18848 (5)	0.0400 (2)	
O1	0.7225 (4)	0.4244 (4)	0.2814 (3)	0.0526 (10)	
O2	0.6104 (4)	0.3689 (4)	0.1814 (3)	0.0488 (10)	
O3	0.6149 (4)	0.6533 (3)	0.2222 (3)	0.0456 (10)	
O4	0.5480 (4)	0.6110 (3)	0.0932 (3)	0.0468 (10)	
O5	0.4181 (4)	0.5712 (3)	0.3508 (3)	0.0456 (9)	
O6	0.3379 (4)	0.5751 (4)	0.2062 (3)	0.0472 (10)	
O7	0.5379 (4)	0.3471 (3)	0.4254 (3)	0.0475 (10)	
O8	0.4080 (4)	0.3342 (3)	0.3120 (3)	0.0471 (10)	
O9	0.6783 (3)	0.5316 (3)	0.4460 (3)	0.0439 (9)	
H91	0.6299	0.5026	0.4969	0.053*	
O10	0.3609 (4)	0.4337 (4)	0.0787 (3)	0.0568 (11)	
H101	0.3857	0.4044	0.0352	0.068*	
C1	0.7095 (6)	0.3671 (5)	0.2257 (4)	0.0469 (14)	
C2	0.8146 (6)	0.2912 (5)	0.2094 (5)	0.0459 (14)	
C3	0.8103 (7)	0.2638 (6)	0.1239 (5)	0.0596 (17)	
H6A	0.7450	0.2945	0.0788	0.072*	
C4	0.9016 (8)	0.1910 (6)	0.1042 (6)	0.076 (2)	
H1A	0.8980	0.1734	0.0463	0.091*	
C5	0.9964 (8)	0.1459 (7)	0.1708 (7)	0.085 (3)	
H16A	1.0579	0.0969	0.1585	0.102*	
C6	1.0021 (7)	0.1721 (7)	0.2555 (7)	0.080 (2)	
H2A	1.0673	0.1395	0.3004	0.095*	
C7	0.9123 (6)	0.2467 (6)	0.2769 (5)	0.0592 (17)	
C8	0.9261 (8)	0.2716 (8)	0.3714 (6)	0.096 (3)	
H86A	1.0008	0.2327	0.4054	0.144*	
H86B	0.8584	0.2429	0.4174	0.144*	
H86C	0.9278	0.3552	0.3519	0.144*	
C9	0.5966 (5)	0.6777 (5)	0.1277 (4)	0.0398 (13)	
C10	0.6395 (5)	0.7941 (5)	0.0541 (4)	0.0418 (13)	
C11	0.7440 (6)	0.8295 (6)	0.0808 (5)	0.0555 (16)	
H12A	0.7828	0.7819	0.1423	0.067*	
C12	0.7921 (7)	0.9338 (6)	0.0185 (6)	0.073 (2)	
H83A	0.8637	0.9561	0.0366	0.088*	
C13	0.7326 (8)	1.0048 (6)	−0.0712 (6)	0.076 (2)	
H15A	0.7642	1.0755	−0.1144	0.091*	
C14	0.6286 (8)	0.9723 (6)	−0.0965 (5)	0.067 (2)	
H29A	0.5886	1.0225	−0.1564	0.080*	
C15	0.5788 (6)	0.8655 (5)	−0.0357 (4)	0.0508 (15)	
C16	0.4611 (7)	0.8351 (7)	−0.0678 (6)	0.078 (2)	
H21A	0.4396	0.7593	−0.0188	0.117*	
H21B	0.4716	0.8329	−0.1347	0.117*	
H21C	0.3974	0.8939	−0.0702	0.117*	
C17	0.3361 (5)	0.6038 (5)	0.2817 (4)	0.0415 (13)	
C18	0.2326 (5)	0.6838 (5)	0.2932 (4)	0.0410 (13)	
C19	0.2546 (6)	0.7553 (5)	0.3447 (5)	0.0556 (16)	
H18A	0.3320	0.7509	0.3709	0.067*	
C20	0.1648 (7)	0.8324 (6)	0.3579 (6)	0.067 (2)	
H4A	0.1816	0.8801	0.3920	0.081*	
C21	0.0502 (7)	0.8382 (6)	0.3201 (6)	0.070 (2)	
H7A	−0.0115	0.8893	0.3291	0.084*	
C22	0.0272 (6)	0.7685 (6)	0.2692 (5)	0.0657 (19)	
H41A	−0.0509	0.7734	0.2441	0.079*	
C23	0.1162 (6)	0.6902 (6)	0.2532 (5)	0.0543 (16)	
C24	0.0820 (7)	0.6149 (8)	0.1987 (6)	0.083 (2)	
H68A	0.1524	0.5665	0.1931	0.124*	
H68B	0.0528	0.6647	0.1311	0.124*	
H68C	0.0191	0.5655	0.2371	0.124*	
C25	0.4550 (5)	0.2973 (5)	0.4010 (4)	0.0442 (14)	
C26	0.4049 (5)	0.1916 (5)	0.4846 (4)	0.0437 (13)	
C27	0.4027 (6)	0.1904 (5)	0.5840 (4)	0.0524 (16)	
H20A	0.4376	0.2499	0.5958	0.063*	
C28	0.3494 (7)	0.1019 (7)	0.6645 (5)	0.070 (2)	
H65A	0.3445	0.1034	0.7298	0.084*	
C29	0.3034 (8)	0.0113 (7)	0.6471 (6)	0.091 (3)	
H61A	0.2679	−0.0493	0.7011	0.109*	
C30	0.3095 (8)	0.0097 (6)	0.5504 (6)	0.081 (2)	
H66A	0.2790	−0.0531	0.5405	0.098*	
C31	0.3604 (6)	0.0998 (6)	0.4662 (5)	0.0580 (17)	
C32	0.3659 (8)	0.0916 (6)	0.3629 (6)	0.079 (2)	
H70A	0.4027	0.1594	0.3134	0.119*	
H70B	0.2847	0.0891	0.3414	0.119*	
H70C	0.4137	0.0212	0.3679	0.119*	
C33	0.8056 (6)	0.5374 (8)	0.4439 (6)	0.073 (2)	
H24A	0.8317	0.5775	0.3729	0.088*	
H24B	0.8434	0.4576	0.4692	0.088*	
C34	0.8510 (6)	0.5986 (6)	0.5052 (5)	0.068 (2)	
H71A	0.9383	0.5985	0.4996	0.102*	
H71B	0.8279	0.5584	0.5761	0.102*	
H71C	0.8162	0.6785	0.4797	0.102*	
C35	0.2514 (6)	0.3709 (6)	0.1090 (5)	0.0588 (17)	
H10A	0.2210	0.3734	0.1734	0.071*	
H10B	0.2706	0.2888	0.1203	0.071*	
C36	0.1552 (7)	0.4221 (7)	0.0298 (6)	0.078 (2)	
H64A	0.0840	0.3780	0.0524	0.117*	
H64B	0.1846	0.4187	−0.0338	0.117*	
H64C	0.1346	0.5029	0.0195	0.117*	

### Atomic displacement parameters (Å^2^)

**Table:**

	*U*^11^	*U*^22^	*U*^33^	*U*^12^	*U*^13^	*U*^23^
Cu1	0.0415 (4)	0.0413 (4)	0.0305 (3)	−0.0075 (3)	−0.0003 (3)	−0.0122 (3)
Cu2	0.0427 (4)	0.0457 (4)	0.0323 (3)	−0.0081 (3)	0.0004 (3)	−0.0153 (3)
O1	0.051 (3)	0.055 (3)	0.058 (3)	−0.006 (2)	0.000 (2)	−0.029 (2)
O2	0.051 (3)	0.054 (2)	0.055 (2)	−0.005 (2)	0.002 (2)	−0.036 (2)
O3	0.059 (3)	0.042 (2)	0.0348 (19)	−0.0204 (19)	0.0013 (18)	−0.0114 (17)
O4	0.061 (3)	0.048 (2)	0.0336 (19)	−0.018 (2)	−0.0009 (18)	−0.0157 (18)
O5	0.047 (2)	0.055 (2)	0.037 (2)	0.0012 (19)	−0.0004 (18)	−0.0210 (18)
O6	0.049 (2)	0.062 (3)	0.0323 (19)	−0.002 (2)	0.0009 (18)	−0.0216 (19)
O7	0.051 (2)	0.045 (2)	0.042 (2)	−0.0176 (19)	−0.0063 (19)	−0.0103 (18)
O8	0.053 (3)	0.046 (2)	0.040 (2)	−0.0239 (19)	0.0015 (19)	−0.0119 (18)
O9	0.047 (2)	0.052 (2)	0.0370 (19)	−0.0150 (19)	0.0031 (18)	−0.0194 (18)
O10	0.053 (3)	0.084 (3)	0.050 (2)	−0.022 (2)	0.007 (2)	−0.042 (2)
C1	0.051 (4)	0.039 (3)	0.043 (3)	−0.012 (3)	0.001 (3)	−0.006 (3)
C2	0.046 (4)	0.036 (3)	0.053 (3)	−0.008 (3)	0.014 (3)	−0.015 (3)
C3	0.071 (5)	0.052 (4)	0.061 (4)	−0.009 (3)	0.020 (3)	−0.028 (3)
C4	0.094 (6)	0.058 (4)	0.079 (5)	−0.009 (4)	0.026 (5)	−0.033 (4)
C5	0.089 (7)	0.061 (5)	0.095 (6)	0.011 (4)	0.034 (5)	−0.027 (5)
C6	0.063 (5)	0.060 (5)	0.097 (6)	0.014 (4)	0.003 (5)	−0.016 (5)
C7	0.054 (4)	0.050 (4)	0.067 (4)	−0.003 (3)	0.006 (3)	−0.017 (3)
C8	0.094 (7)	0.110 (7)	0.088 (6)	0.026 (5)	−0.037 (5)	−0.046 (6)
C9	0.039 (3)	0.044 (3)	0.032 (3)	0.000 (3)	0.004 (2)	−0.012 (2)
C10	0.049 (3)	0.038 (3)	0.035 (3)	−0.006 (3)	0.001 (3)	−0.010 (2)
C11	0.061 (4)	0.049 (4)	0.052 (4)	−0.019 (3)	0.001 (3)	−0.013 (3)
C12	0.079 (5)	0.066 (5)	0.071 (5)	−0.030 (4)	0.011 (4)	−0.021 (4)
C13	0.106 (7)	0.050 (4)	0.063 (5)	−0.027 (4)	0.024 (5)	−0.011 (4)
C14	0.097 (6)	0.046 (4)	0.046 (4)	−0.005 (4)	0.004 (4)	−0.007 (3)
C15	0.065 (4)	0.043 (3)	0.043 (3)	0.001 (3)	0.002 (3)	−0.017 (3)
C16	0.082 (6)	0.072 (5)	0.070 (5)	0.004 (4)	−0.026 (4)	−0.017 (4)
C17	0.042 (3)	0.042 (3)	0.031 (3)	−0.008 (3)	0.001 (2)	−0.004 (2)
C18	0.043 (3)	0.037 (3)	0.037 (3)	−0.008 (3)	0.003 (3)	−0.007 (2)
C19	0.063 (4)	0.049 (4)	0.059 (4)	−0.006 (3)	0.006 (3)	−0.025 (3)
C20	0.086 (6)	0.047 (4)	0.076 (5)	−0.003 (4)	0.011 (4)	−0.031 (4)
C21	0.062 (5)	0.060 (4)	0.078 (5)	0.011 (4)	0.009 (4)	−0.020 (4)
C22	0.047 (4)	0.074 (5)	0.070 (5)	0.007 (4)	−0.009 (3)	−0.023 (4)
C23	0.051 (4)	0.059 (4)	0.048 (3)	−0.002 (3)	−0.002 (3)	−0.016 (3)
C24	0.057 (5)	0.116 (7)	0.091 (6)	0.004 (5)	−0.022 (4)	−0.057 (5)
C25	0.048 (4)	0.046 (3)	0.034 (3)	0.001 (3)	0.007 (3)	−0.012 (3)
C26	0.043 (3)	0.033 (3)	0.047 (3)	−0.005 (2)	0.001 (3)	−0.007 (3)
C27	0.061 (4)	0.045 (3)	0.041 (3)	−0.003 (3)	0.003 (3)	−0.008 (3)
C28	0.073 (5)	0.067 (5)	0.049 (4)	−0.007 (4)	0.012 (4)	−0.002 (3)
C29	0.097 (7)	0.068 (5)	0.072 (5)	−0.031 (5)	0.008 (5)	0.014 (4)
C30	0.094 (6)	0.055 (4)	0.084 (6)	−0.038 (4)	−0.007 (5)	−0.009 (4)
C31	0.057 (4)	0.047 (4)	0.059 (4)	−0.014 (3)	−0.008 (3)	−0.008 (3)
C32	0.099 (6)	0.056 (4)	0.083 (5)	−0.016 (4)	−0.017 (5)	−0.025 (4)
C33	0.042 (4)	0.117 (7)	0.086 (5)	−0.023 (4)	0.008 (4)	−0.065 (5)
C34	0.057 (4)	0.075 (5)	0.075 (5)	−0.017 (4)	−0.012 (4)	−0.029 (4)
C35	0.059 (4)	0.066 (4)	0.053 (4)	−0.008 (3)	−0.004 (3)	−0.024 (3)
C36	0.058 (5)	0.078 (5)	0.084 (5)	−0.014 (4)	−0.013 (4)	−0.017 (4)

### Geometric parameters (Å, °)

**Table:**

Cu1—O7	1.928 (4)	C14—H29A	0.9300
Cu1—O3	1.952 (4)	C15—C16	1.509 (9)
Cu1—O1	1.975 (4)	C16—H21A	0.9600
Cu1—O5	2.017 (4)	C16—H21B	0.9600
Cu1—O9	2.149 (4)	C16—H21C	0.9600
Cu1—Cu2	2.6005 (11)	C17—C18	1.495 (8)
Cu2—O2	1.931 (4)	C18—C19	1.389 (8)
Cu2—O6	1.954 (4)	C18—C23	1.402 (8)
Cu2—O8	1.993 (4)	C19—C20	1.378 (9)
Cu2—O4	2.008 (4)	C19—H18A	0.9300
Cu2—O10	2.161 (4)	C20—C21	1.372 (10)
O1—C1	1.269 (7)	C20—H4A	0.9300
O2—C1	1.271 (7)	C21—C22	1.367 (10)
O3—C9	1.269 (6)	C21—H7A	0.9300
O4—C9	1.270 (6)	C22—C23	1.397 (9)
O5—C17	1.275 (6)	C22—H41A	0.9300
O6—C17	1.252 (6)	C23—C24	1.500 (9)
O7—C25	1.268 (7)	C24—H68A	0.9600
O8—C25	1.271 (6)	C24—H68B	0.9600
O9—C33	1.405 (7)	C24—H68C	0.9600
O9—H91	0.8523	C25—C26	1.498 (7)
O10—C35	1.442 (7)	C26—C31	1.389 (8)
O10—H101	0.8558	C26—C27	1.399 (8)
C1—C2	1.500 (8)	C27—C28	1.378 (8)
C2—C3	1.382 (8)	C27—H20A	0.9300
C2—C7	1.391 (9)	C28—C29	1.374 (11)
C3—C4	1.389 (9)	C28—H65A	0.9300
C3—H6A	0.9300	C29—C30	1.376 (11)
C4—C5	1.361 (11)	C29—H61A	0.9300
C4—H1A	0.9300	C30—C31	1.401 (9)
C5—C6	1.363 (11)	C30—H66A	0.9300
C5—H16A	0.9300	C31—C32	1.503 (10)
C6—C7	1.405 (10)	C32—H70A	0.9600
C6—H2A	0.9300	C32—H70B	0.9600
C7—C8	1.501 (10)	C32—H70C	0.9600
C8—H86A	0.9600	C33—C34	1.479 (9)
C8—H86B	0.9600	C33—H24A	0.9700
C8—H86C	0.9600	C33—H24B	0.9700
C9—C10	1.502 (7)	C34—H71A	0.9600
C10—C11	1.378 (8)	C34—H71B	0.9600
C10—C15	1.385 (8)	C34—H71C	0.9600
C11—C12	1.375 (9)	C35—C36	1.482 (9)
C11—H12A	0.9300	C35—H10A	0.9700
C12—C13	1.377 (10)	C35—H10B	0.9700
C12—H83A	0.9300	C36—H64A	0.9600
C13—C14	1.347 (10)	C36—H64B	0.9600
C13—H15A	0.9300	C36—H64C	0.9600
C14—C15	1.400 (9)		
			
O7—Cu1—O3	172.78 (16)	C15—C14—H29A	118.9
O7—Cu1—O1	90.04 (18)	C10—C15—C14	117.3 (6)
O3—Cu1—O1	90.54 (18)	C10—C15—C16	123.1 (6)
O7—Cu1—O5	89.64 (17)	C14—C15—C16	119.5 (6)
O3—Cu1—O5	87.92 (17)	C15—C16—H21A	109.5
O1—Cu1—O5	164.85 (16)	C15—C16—H21B	109.5
O7—Cu1—O9	93.24 (15)	H21A—C16—H21B	109.5
O3—Cu1—O9	93.82 (15)	C15—C16—H21C	109.5
O1—Cu1—O9	97.89 (16)	H21A—C16—H21C	109.5
O5—Cu1—O9	97.25 (15)	H21B—C16—H21C	109.5
O7—Cu1—Cu2	87.80 (12)	O6—C17—O5	124.8 (5)
O3—Cu1—Cu2	85.12 (11)	O6—C17—C18	119.1 (5)
O1—Cu1—Cu2	82.78 (12)	O5—C17—C18	116.1 (5)
O5—Cu1—Cu2	82.07 (11)	C19—C18—C23	119.4 (6)
O9—Cu1—Cu2	178.76 (11)	C19—C18—C17	117.8 (5)
O2—Cu2—O6	171.58 (17)	C23—C18—C17	122.8 (6)
O2—Cu2—O8	90.35 (18)	C20—C19—C18	121.7 (7)
O6—Cu2—O8	90.75 (17)	C20—C19—H18A	119.1
O2—Cu2—O4	90.02 (18)	C18—C19—H18A	119.1
O6—Cu2—O4	86.69 (17)	C21—C20—C19	119.3 (7)
O8—Cu2—O4	164.46 (15)	C21—C20—H4A	120.4
O2—Cu2—O10	96.24 (17)	C19—C20—H4A	120.4
O6—Cu2—O10	91.96 (17)	C22—C21—C20	119.7 (7)
O8—Cu2—O10	95.56 (16)	C22—C21—H7A	120.2
O4—Cu2—O10	99.84 (16)	C20—C21—H7A	120.2
O2—Cu2—Cu1	85.76 (12)	C21—C22—C23	122.8 (7)
O6—Cu2—Cu1	86.17 (11)	C21—C22—H41A	118.6
O8—Cu2—Cu1	80.87 (11)	C23—C22—H41A	118.6
O4—Cu2—Cu1	83.67 (11)	C22—C23—C18	117.2 (6)
O10—Cu2—Cu1	175.93 (12)	C22—C23—C24	119.1 (6)
C1—O1—Cu1	122.8 (4)	C18—C23—C24	123.6 (6)
C1—O2—Cu2	122.0 (4)	C23—C24—H68A	109.5
C9—O3—Cu1	122.4 (4)	C23—C24—H68B	109.5
C9—O4—Cu2	121.0 (3)	H68A—C24—H68B	109.5
C17—O5—Cu1	121.7 (4)	C23—C24—H68C	109.5
C17—O6—Cu2	121.4 (4)	H68A—C24—H68C	109.5
C25—O7—Cu1	119.6 (3)	H68B—C24—H68C	109.5
C25—O8—Cu2	124.4 (4)	O7—C25—O8	124.5 (5)
C33—O9—Cu1	122.3 (4)	O7—C25—C26	117.4 (5)
C33—O9—H91	130.1	O8—C25—C26	118.0 (5)
Cu1—O9—H91	99.7	C31—C26—C27	120.7 (5)
C35—O10—Cu2	121.6 (3)	C31—C26—C25	123.0 (5)
C35—O10—H101	96.1	C27—C26—C25	116.3 (5)
Cu2—O10—H101	128.2	C28—C27—C26	120.7 (6)
O1—C1—O2	124.0 (6)	C28—C27—H20A	119.7
O1—C1—C2	120.0 (6)	C26—C27—H20A	119.7
O2—C1—C2	116.0 (6)	C29—C28—C27	119.1 (7)
C3—C2—C7	120.0 (6)	C29—C28—H65A	120.4
C3—C2—C1	117.1 (6)	C27—C28—H65A	120.4
C7—C2—C1	122.9 (6)	C28—C29—C30	120.4 (7)
C2—C3—C4	121.2 (7)	C28—C29—H61A	119.8
C2—C3—H6A	119.4	C30—C29—H61A	119.8
C4—C3—H6A	119.4	C29—C30—C31	121.9 (7)
C5—C4—C3	119.0 (8)	C29—C30—H66A	119.0
C5—C4—H1A	120.5	C31—C30—H66A	119.0
C3—C4—H1A	120.5	C26—C31—C30	117.1 (7)
C4—C5—C6	120.6 (8)	C26—C31—C32	123.7 (6)
C4—C5—H16A	119.7	C30—C31—C32	119.2 (6)
C6—C5—H16A	119.7	C31—C32—H70A	109.5
C5—C6—C7	121.9 (8)	C31—C32—H70B	109.5
C5—C6—H2A	119.0	H70A—C32—H70B	109.5
C7—C6—H2A	119.0	C31—C32—H70C	109.5
C2—C7—C6	117.3 (7)	H70A—C32—H70C	109.5
C2—C7—C8	124.2 (6)	H70B—C32—H70C	109.5
C6—C7—C8	118.5 (7)	O9—C33—C34	114.9 (6)
C7—C8—H86A	109.5	O9—C33—H24A	108.6
C7—C8—H86B	109.5	C34—C33—H24A	108.6
H86A—C8—H86B	109.5	O9—C33—H24B	108.6
C7—C8—H86C	109.5	C34—C33—H24B	108.6
H86A—C8—H86C	109.5	H24A—C33—H24B	107.5
H86B—C8—H86C	109.5	C33—C34—H71A	109.5
O3—C9—O4	124.9 (5)	C33—C34—H71B	109.5
O3—C9—C10	115.2 (5)	H71A—C34—H71B	109.5
O4—C9—C10	119.8 (5)	C33—C34—H71C	109.5
C11—C10—C15	120.1 (5)	H71A—C34—H71C	109.5
C11—C10—C9	116.5 (5)	H71B—C34—H71C	109.5
C15—C10—C9	123.5 (5)	O10—C35—C36	111.8 (5)
C12—C11—C10	121.3 (6)	O10—C35—H10A	109.3
C12—C11—H12A	119.3	C36—C35—H10A	109.3
C10—C11—H12A	119.3	O10—C35—H10B	109.3
C11—C12—C13	118.9 (7)	C36—C35—H10B	109.3
C11—C12—H83A	120.6	H10A—C35—H10B	107.9
C13—C12—H83A	120.6	C35—C36—H64A	109.5
C14—C13—C12	120.2 (7)	C35—C36—H64B	109.5
C14—C13—H15A	119.9	H64A—C36—H64B	109.5
C12—C13—H15A	119.9	C35—C36—H64C	109.5
C13—C14—C15	122.3 (7)	H64A—C36—H64C	109.5
C13—C14—H29A	118.9	H64B—C36—H64C	109.5

### Hydrogen-bond geometry (Å, °)

**Table:**

*D*—H···*A*	*D*—H	H···*A*	*D*···*A*	*D*—H···*A*
O9—H91···O5^i^	0.85	2.04	2.841 (6)	156
O10—H101···O4^ii^	0.86	2.00	2.831 (6)	162

Symmetry codes: (i) −*x*+1, −*y*+1, −*z*+1; (ii) −*x*+1, −*y*+1, −*z*.

## References

[bb1] Danish, M., Saleem, I., Tahir, M. N., Ahmad, N. & Raza, A. R. (2010). *Acta Cryst.* E**66**, m528. 10.1107/S1600536810013322 PMC2979172 21579022

[bb2] Higashi, T. (1995). *ABSCOR* Rigaku Corporation, Tokyo, Japan.

[bb3] Melnik, M., Dunaj Jurco, M. & Handlovic, M. (1984). *Inorg. Chim. Acta*, **86**, 185–190.

[bb4] Rigaku (1998). *RAPID-AUTO* Rigaku Corporation, Tokyo, Japan.

[bb5] Rigaku/MSC (2004). *CrystalStructure* Rigaku/MSC Inc., The Woodlands, Texas, USA.

[bb6] Sheldrick, G. M. (2008). *Acta Cryst.* A**64**, 112–122. 10.1107/S0108767307043930 18156677

[bb7] Sunil, A. C., Bezuidenhoudt, B. C. B. & Janse van Rensburg, J. M. (2008). *Acta Cryst.* E**64**, m553–m554. 10.1107/S1600536808006661 PMC2960998 21202010

